# Harmonizing Newborn Screening Laboratory Proficiency Test Results Using the CDC NSQAP Reference Materials

**DOI:** 10.3390/ijns6030075

**Published:** 2020-09-17

**Authors:** Charles Austin Pickens, Maya Sternberg, Mary Seeterlin, Víctor R. De Jesús, Mark Morrissey, Adrienne Manning, Sonal Bhakta, Patrice K. Held, Joanne Mei, Carla Cuthbert, Konstantinos Petritis

**Affiliations:** 1 Centers for Disease Control and Prevention, Division of Laboratory Sciences, Newborn Screening and Molecular Biology Branch, MS F19, Atlanta, GA 30341, USA; ogh6@cdc.gov (C.A.P.); mrs7@cdc.gov (M.S.); jvm0@cdc.gov (J.M.); ijz6@cdc.gov (C.C.); 2 Michigan Department of Community Health, Lansing, MI 49221, USA; SeeterlinM@michigan.gov; 3 Centers for Disease Control and Prevention, Division of Laboratory Sciences, Tobacco and Volatiles Branch, Atlanta, GA 30341, USA; FOA5@cdc.gov; 4 Wadsworth Center/New York State Department of Health, Albany, NY 12201-0509, USA; mark.morrissey@health.ny.gov; 5 Katherine A. Kelley State Public Health Laboratory, Connecticut Department of Public Health, Rocky Hill, CT 06067, USA; Adrienne.Manning@ct.gov; 6 Arizona Department of Health Services, Office of Newborn Screening, Phoenix, AZ 85007, USA; sonal.bhakta@azdhs.gov; 7 Wisconsin State Laboratory of Hygiene, University of Wisconsin School of Medicine and Public Health, Madison, WI 53726, USA; patrice.held@slh.wisc.edu

**Keywords:** mass spectrometry, normalization, newborn screening, proficiency testing, metabolite

## Abstract

Newborn screening (NBS) laboratories cannot accurately compare mass spectrometry-derived results and cutoff values due to differences in testing methodologies. The objective of this study was to assess harmonization of laboratory proficiency test (PT) results using quality control (QC) data. Newborn Screening Quality Assurance Program (NSQAP) QC and PT data reported from 302 laboratories in 2019 were used to compare results among laboratories. QC materials were provided as dried blood spot cards which included a base pool and the base pool enriched with specific concentrations of metabolites in a linear range. QC data reported by laboratories were regressed on QC data reported by the Centers for Disease Control and Prevention (CDC), and laboratory’s regression parameters were used to harmonize their PT result. In general, harmonization tended to reduce overall variation in PT data across laboratories. The metabolites glutarylcarnitine (C5DC), tyrosine, and phenylalanine were displayed to highlight inter- and intra-method variability in NBS results. Several limitations were identified using retrospective data for harmonization, and future studies will address these limitations to further assess feasibility of using NSQAP QC data to harmonize PT data. Harmonizing NBS data using common QC materials appears promising to aid result comparison between laboratories.

## 1. Introduction

Newborn screening (NBS) aims to identify newborns at risk of inborn errors of metabolism (IEM), such as amino acid, organic acid, and fatty acid oxidation metabolic disorders. Routine NBS uses flow injection analysis tandem mass spectrometry (FIA-MS/MS) to screen several dozen metabolites associated with over thirty disorders from a newborn’s dried blood spot (DBS) [1,2]. Mass spectrometry-based NBS quantifies metabolites through either derivatized or non-derivatized protocols via laboratory developed tests (LDT) or commercial kits as previously described by Chace et al. [3]. When specific metabolites in a newborn’s DBS exceed laboratory-specific cutoffs, the newborn is subjected to follow-up testing [4]. Since laboratory testing methodologies that establish cutoffs vary, it is difficult to directly compare metabolite results and cutoffs between NBS laboratories. Variability between NBS results and cutoffs may also occur for the following reasons: not accounting for metabolite recovery, the use of additional metabolites or metabolite ratios per screening disorder [5,6,7,8,9,10,11], differences in mass spectrometer vendor and model, differences in internal standard surrogates [12,13], and varying use of calibration curves.

The Centers for Disease Control and Prevention (CDC) enhances quality and maintains accuracy of NBS through the Newborn Screening Quality Assurance Program (NSQAP). The NSQAP provides quality assurance (QA) services to participating laboratories, such as technical guidance and proficiency testing (PT) specimens that mimic metabolite concentrations of newborns with metabolic disorders [14]. These PT materials help laboratories meet QA testing accuracy verification requirements, and to assess their ability to correctly classify presumptive positive and normal samples during routine NBS [2]. In addition to PT specimens, the NSQAP provides quality control (QC) materials so laboratories can periodically manage and verify the overall quality of laboratory testing. Despite participants receiving homogenous NSQAP QC and PT materials, reported results often differ several fold due to methodological differences employed during DBS analysis, and because most NBS laboratories do not apply correction factors to account for metabolite recovery. Therefore, comparison of metabolite values and cutoffs between laboratories is difficult.

Currently, NBS cutoffs are laboratory-specific since population demographics and analytical testing methodologies influence metabolite thresholds that classify samples as normal or presumptive positive. This is particularly true for IEM since many of these disorders are very rare. For example, geographical regions containing consanguineous populations may have certain genetic variants [15,16,17] associated with an IEM. Since diseases are continually added to the Recommended Uniform Screening Panel [18], laboratories may begin screening for a disease with no positive specimens to aid cutoff establishment. Therefore, harmonization of analytical methodologies may yield comparable metabolite measurements and assist in laboratory cutoff determination. The Clinical and Laboratory Standards Institute defines harmonization as “the process of recognizing, understanding, and explaining differences while taking steps to achieve uniformity of results, or at minimum, a means of conversion of results such that different groups can use the data obtained from assays interchangeably” [19].

Discrepancies in clinical metabolite measurements are not unique to NBS, and overall there is a demand for method or result harmonization. The major advantage of harmonized test results in laboratory assays include the use of common decision limits specified in clinical guidelines across all methods and the uniform interpretation of results [20,21,22,23]. Several studies have investigated or proposed strategies to compare inter-laboratory results and assess variation using certified reference materials (e.g., PT and QC materials) in combination with a reference laboratory [24,25,26,27]. Recent work demonstrated that QC samples could be used to standardize MS results across laboratories using the same extraction protocol and type of mass spectrometer [28]. However, NBS laboratories use a variety of sample extraction protocols and mass spectrometers. Despite the heightened interest for harmonization of inter-laboratory MS results, there have been few attempts applied to NBS [29,30,31]. 

In 2016, several US public health NBS laboratories, in collaboration with the CDC, conducted a pilot study to investigate the use of NSQAP QC materials to harmonize their metabolite cutoff and PT results [32]. These data [32] demonstrated potential for correcting methodological differences and decreasing inter-laboratory variation associated with PT results. Such corrections utilized calibration curves constructed with NSQAP QC values generated by the CDC’s Biochemical Mass Spectrometry Laboratory (hereafter referred to as CDC). Since the NSQAP provides both QC and PT materials, along with collecting results from participating laboratories, there is a unique opportunity to assess the inter-laboratory variation between raw and harmonized PT values using the QC materials. To our knowledge, this is the first study to use retrospective QC results to harmonize PT data reported across NBS laboratories. 

## 2. Methods 

**Figure 1:**
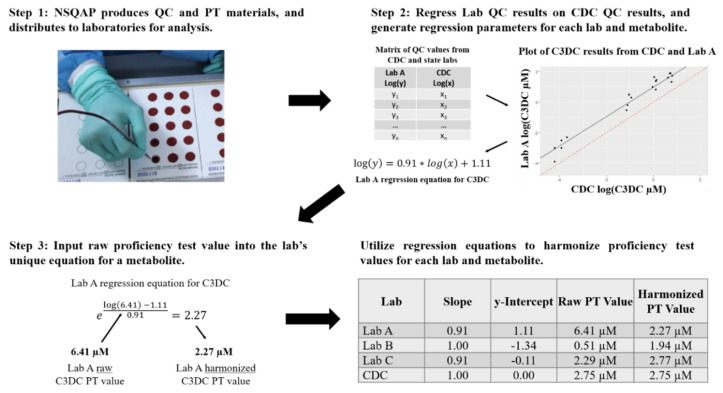
Overview of Newborn Screening Quality Assurance Program quality control and proficiency test material production to data harmonization. The Center for Disease Control and Prevention (CDC) Newborn Screening Quality Assurance Program (NSQAP) quality control (QC) and proficiency test (PT) materials were produced and shipped to participating laboratories. Laboratories reported their QC and PT data back to NSQAP. For each metabolite, laboratories reported QC quantified values from four QC pools acquired from five independent runs, along with single PT specimen measurements. Reported QC data were then regressed on QC data reported from the CDC Biochemical Mass Spectrometry Laboratory. The regression generated a unique equation for a laboratory and an associated metabolite. PT data reported from each laboratory were then input into their unique regression equation to achieve a harmonized PT value. C3DC, Malonylcarnitine; Log, Natural logarithm.

Appropriate safety control measures (including engineering, administrative policy and procedure, and personal protective equipment) were used for all procedures based on a site-specific risk assessment that identified physical, health, and procedural hazards. The study used NSQAP QC and PT data reported by 302 laboratories in 2019. QC and PT materials were provided as DBS blood collection cards, which are the common sample type for NBS specimens [33]. The QC DBS cards included a base pool, nonenriched hematocrit-adjusted blood, and three linearly enriched versions of the base pool including all metabolites in Table 1. The expected metabolite values in DBS materials were calculated by summing the endogenous concentration of each metabolite in the blood and the concentration of each corresponding metabolite enriched into the blood. Additional information regarding pool preparation and concentrations can be found in the NSQAP 2019 Quality Control Program Report [34]. An overview of our study from QC and PT material production to data harmonization is summarized in Figure 1. After production in the NSQAP laboratories, QC materials and PT panels were routinely shipped to NBS laboratories. PT panels consisted of five-blinded coded specimens of one DBS per specimen. Participating laboratories were requested to run each of the four QC pools in duplicate in five independent runs, totaling 40 measurements for each metabolite. Laboratories were instructed to prepare and analyze each PT specimen as they would a newborn DBS specimen. QC and PT data were submitted by the data reporting deadlines.

QC and PT materials were shipped in 2019 in quarters one and three; and PT materials were shipped in quarter four. Labs are instructed to analyze PT specimens’ data within one month from the shipment date. The frequency of QC specimen analysis could vary from consecutive days to several days or weeks apart. QC results were due about 3 months after the shipping date. NSQAP does not collect dates of analysis. As a result, PT and QC materials may have been run within a few days of each other or up to four months apart. NSQAP collects the type of method used for analysis by the laboratory, but does not track specific instrument types. We are aware of instances where laboratories analyzed their QC and PT materials using different instruments in their lab.

The duplicate QC concentrations for each day were averaged, totaling 20 measurements for each lab and metabolite. These averaged QC values were used to fit a simple linear regression model separately, for each laboratory and metabolite. Assumptions for a linear regression model include independent and identically distributed normal errors with a mean of zero and constant variance. Since the QC pools are enriched with metabolites from low to high across the pools, the pool standard deviation tended to be proportional to the mean. A natural logarithmic transformation was used to stabilize the variance, herein natural logarithm will be referred to as log. Therefore, the log-transformed QC metabolite concentrations reported from a laboratory for a specific metabolite were regressed on the log-transformed QC metabolite concentrations reported from CDC for the same metabolite. In regression models, QC values were paired by day of analysis between each lab and CDC for convenience. For each metabolite, the single PT specimen concentration submitted by each laboratory was input into the appropriate regression equation and solved to provide a harmonized PT value for each metabolite as outlined in Figure 1.

All laboratory results and their method data reported in this study are anonymized. For each metabolite, laboratories that did not submit QC materials were omitted, because QC data are required to construct regression lines required for PT harmonization. Furthermore, laboratories that did not report PT values for a metabolite were also excluded from the analysis since PT values were required to assess harmonization. Over 50% of laboratories reported PT data from metabolites palmitoylcarnitine (C16), hydroxybutyrylcarnitine (C4OH), propionylcarnitine (C3), and succinylacetone (SUAC) that exceeded their highest QC value, thus, harmonized results were extrapolated outside their linear range. All statistical analyses were conducted using R v 3.6.2 [35].

## 3. Results

### 3.1. Metabolite and Specimen Descriptive Statistics

This study focused on data reported by US public health NBS laboratories; however, data used for phenylalanine also included US non-public health and international NBS laboratories to demonstrate harmonization across a large number of methods and laboratories. Table 1 presents 27 metabolites and their expected values, CDC PT results, and the average and range of raw PT results obtained by the participating laboratories. Two separate PT programs were administered for amino acids and acylcarnitines, since some acylcarnitines can only be distinguished by derivatization under FIA with low-resolving MS/MS. For the metabolites and specimen identifiers in Table 1, all amino acid data, octanoylcarnitine (C8), and stearoylcarnitine (C18) were harmonized to the CDC’s non-derivatized method, while the remaining acylcarnitines were harmonized to the CDC derivatized method [36]. The range of raw PT values highlight the variability in results despite using the same homogenous specimen. Much higher variability was observed for the phenylalanine data as it includes a larger number of analytical methods and laboratories (i.e., US public health, US non-public health, and international NBS laboratories). Figure S1 presents the distribution of regression equation slopes and intercepts across the metabolites and laboratories, to visualize the dispersion of data across participating laboratories. Additional information on how regression parameters influence harmonized PT values is also included.

### 3.2. Visualization of Raw and Harmonized Proficiency Values

**Figure 2:**
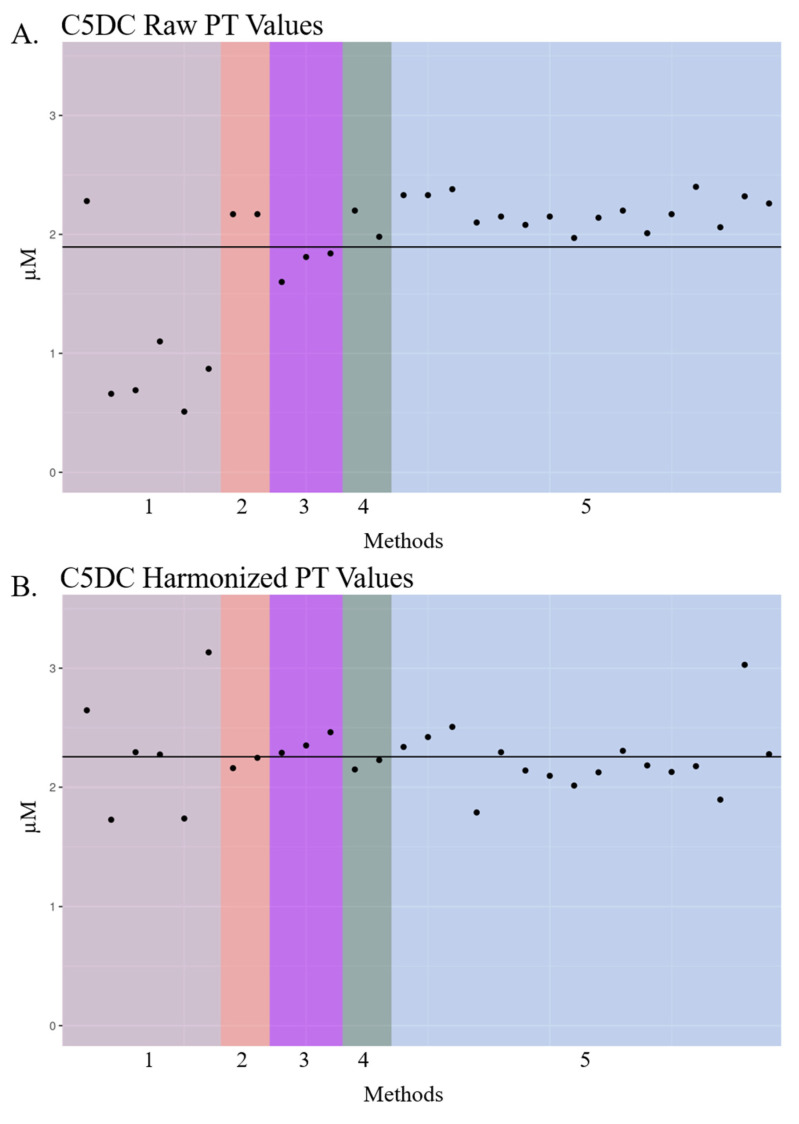
Dot plot of glutarylcarnitine (C5DC) proficiency test raw and harmonized data across US public health newborn screening (NBS) labs from 2019. Dot plots of raw (**A**,**B**) harmonized glutarylcarnitine (C5DC) proficiency test (PT) data reported by US public health NBS laboratories in 2019. Laboratory names are de-identified. Laboratory PT values are grouped by analytical method, and these unique methods are denoted by color and number. Each “•” indicates a unique US public health NBS laboratory’s PT concentration for C5DC. The black solid line represents the mean of PT values in each plot. Method 1: Derivatized—MS/MS LDT; Method 2: PerkinElmer NeoGram AAAC Tandem Mass Spectrometry kit (Waltham, MA, USA); Method 3: Perkin Elmer Neobase^TM^ 2 Non-derivatized MSMS kit (Waltham, MA, USA); Method 4: Non-derivatized—MS/MS LDT; Method 5: Perkin Elmer Neobase^TM^ Non-derivatized MSMS kit (Waltham, MA, USA).

Several metabolites displayed in Figure 2A,B highlight the differences in laboratory-reported PT values when different methods are employed. For example, glutarylcarnitine (C5DC) raw PT values often differed depending on the NBS laboratory method (Figure 2A,B), and most laboratories using Derivatized—MS/MS LDT (Method 1) reported lower concentrations of C5DC 1 µM than other methods. Otherwise, the average raw PT value across methods was close to the expected value of 1.82 µM. After harmonization, there was less variation across the methods with more uniformity around the mean (Figure 2B). In line with historical data reported to NSQAP (not shown), the mean of C5DC PT results was above the expected value along with the mean of harmonized C5DC PT values (Table 1). PT specimens containing enriched C5DC did not contain detectable amounts of C6OH, as confirmed by high-resolution mass spectrometry analysis [37], which cannot be separated in non-derivatized methods using triple quadrupole mass spectrometry platforms. After contacting several laboratories, it was determined that laboratories with apparent harmonized PT outliers (Figure 2A,B) typically ran their QC materials and PT materials on different instruments. The acquisition of QC and PT data on different instruments would account for the larger variability in their harmonized PT values, across metabolites in our study, when compared with the harmonized PT values of the other laboratories. NBS laboratories are accustomed to obtaining different quantitative values for the same metabolite/specimen when acquired on different vendor mass spectrometers in their laboratories, and even on the same vendor’s instruments, which is why laboratories often have cutoffs specific to each instrument. 

**Figure 3:**
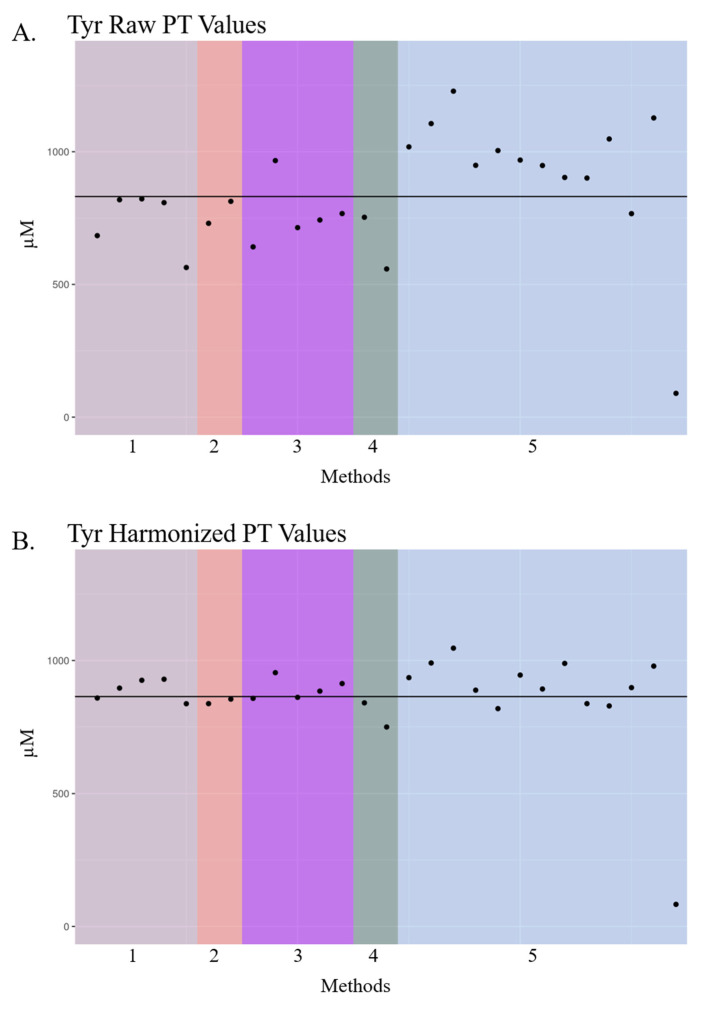
Dot plot of tyrosine proficiency test raw and harmonized data across US public health NBS labs from 2019. Dot plots of raw (**A**,**B**) harmonized tyrosine (Tyr) proficiency test (PT) data reported by US public health NBS laboratories in 2019. Laboratory names are de-identified. Laboratory PT values are grouped by analytical method, and these unique methods are denoted by color and number. Each “•” indicates a unique state laboratory’s PT concentration for Tyr. The black solid line represents the mean of PT values in each plot. Method 1: Derivatized—MS/MS LDT; Method 2: PerkinElmer NeoGram AAAC Tandem Mass Spectrometry kit (Waltham, MA, USA); Method 3: Perkin Elmer Neobase^TM^ 2 Non-derivatized MSMS kit (Waltham, MA, USA); Method 4: Non-derivatized—MS/MS LDT; Method 5: PerkinElmer NeoBase^TM^ Non-derivatized MSMS Kit (Waltham, MA, USA).

**Figure 4:**
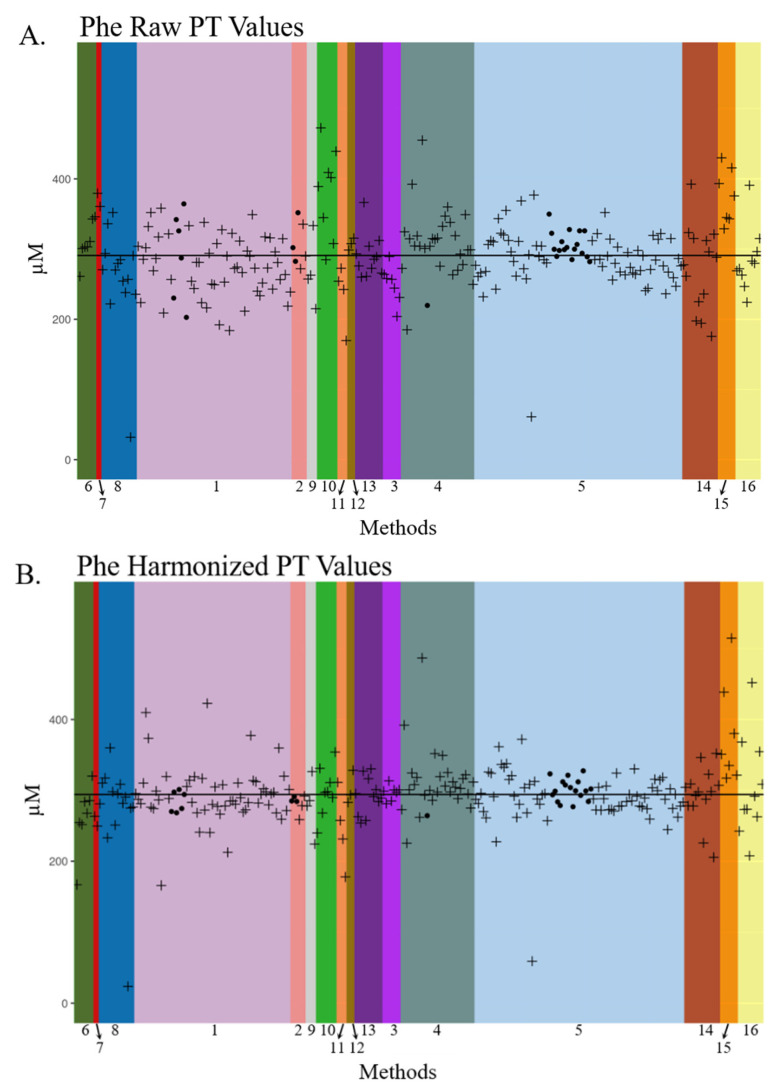
Dot plot of phenylalanine proficiency test raw and harmonized data across participating newborn screening labs from 2019. Dot plots of raw (**A**,**B**) harmonized phenylalanine (Phe) proficiency test (PT) data reported by US public health and international NBS laboratories in 2019. Laboratory names are de-identified. Laboratory PT values are grouped by analytical method, and these unique methods are denoted by color and number. Each “•” indicates a unique US public health laboratory’s PT concentration and each “**+**” indicates a unique US non-public health or international laboratory’s PT concentration for Phe. The black solid lines represent the mean of PT values in each plot. Method 1: Derivatized—MS/MS LDT; Method 2: PerkinElmer NeoGram AAAC Tandem Mass Spectrometry kit (Waltham, MA, USA); Method 3: Perkin Elmer Neobase^TM^ 2 Non-derivatized MSMS kit (Waltham, MA, USA); Method 4: Non-derivatized - MS/MS LDT; Method 5: PerkinElmer NeoBase AAAC Tandem Mass Spectrometry kit (Waltham, MA, USA); Method 6: Labsystems Neonatal Phenylalanine (Vantaa, Finland); Method 7: Quantase Phenylalanine Screening Assay (Hercules, CA, USA); Method 8: MassChrom^®^ Amino Acids and Acylcarnitines from Dried Blood—LC-MS/MS (Gräfelfing, Germany); Method 9: RECIPE ClinSpot^®^ LC−MS/MS Complete Kit (München, Germany); Method 10: Fluorometric manual; Method 11: Interscientific Enzyme (Hollywood, FL, USA); Method 12: MS2 Screening Neo (MS−Neo) Siemens (Tokyo, Japan); Method 13: MassChrom^®^ Amino Acids and Acylcarnitines from Dried Blood/Non Derivatised—LC-MS/MS (Gräfelfing, Germany); Method 14: Other; Method 15: PerkinElmer GSP Neonatal Phenylalanine kit (Waltham, MA, USA); Method 16: PerkinElmer Neonatal Phenylalanine kit (Waltham, MA, USA).

Tyrosine (Tyr) raw PT results also varied by method with concentrations deviating over 300 µM above and below the expected value (Figure 3A). Nearly all labs reported Tyr concentrations lower than the expected value of 900 µM, except those employing Method 5. The variation of the PT values post-harmonization was smaller, and the deviations of the laboratory results from the mean were distributed more uniformly across all methods (Figure 3B). Phenylalanine (Phe) dot plots presented in Figure 4 include US public health, US non-public health, and international NBS laboratories using MS/MS LDT and commercial kits, along with in-house or commercial non-MS/MS methods such as fluorescent, colorimetric, and enzymatic assays (Figure 4). There was a large amount of inter- and intra-method variation in raw Phe PT data across the 268 laboratories using 16 methods. After harmonization, Phe data became more similar across nearly all methods, with the inter-laboratory standard deviation decreasing from 52 in raw PT values to 46 in harmonized PT values (data not shown), thus demonstrating the potential for harmonizing MS/MS- and non-MS/MS-acquired data.

### 3.3. Study Limitations

The purpose of this study was to investigate method harmonization using NSQAP QC and PT data reported by participating NBS laboratories and the CDC as a reference laboratory. We acknowledge several limitations in our current study. The PT and QC programs were not initially designed for harmonization and neither were the NBS laboratory data reported back to the NSQAP. PT harmonization using QC values requires that QC and PT data are acquired on the same analytical instrument. Even though harmonization of PT results appeared to mitigate method differences, there were several outlier laboratories in post-harmonized data. After contacting several laboratories, we became aware that some laboratories acquire QC and PT data on different mass spectrometers, thus elucidating why their PT results appear as outliers post-harmonization. In addition, our current QC and PT data reporting system requires manual data entry. Although we screen for outliers and systematic errors in reported data, it is possible NBS laboratories could have entered incorrect QC data values, which would impact their regression slopes and their harmonized PT values. 

Furthermore, NBS laboratories often reported PT values that exceeded their reported maximum QC values for some metabolites. In other words, their raw PT values were outside of their harmonization curve, which may have introduced error into their harmonized PT value. Based on these limitations, we modified our future QC material productions to enhance method harmonization by enriching metabolites that are frequently reported as zero and increasing the maximum QC concentration of several metabolites. For convenience, QC values were paired by day of analysis during regression analyses between CDC and each laboratory, thus, different orders of QC pairing would result in slightly different regression parameters and harmonized PT concentrations. 

The purpose of PT is to simulate the analysis of clinical specimens by participating laboratories [38]. Since laboratories only submit one value for metabolites in each PT specimen, we were unable to estimate the uncertainty in raw and harmonized PT values using the regression method employed in the pilot study and our current study [32]. Lastly, the harmonization method presented in this study requires the reference laboratory to have an accurate method with minimal to no bias. QC data reported by CDC were acquired using an FIA-MS/MS method that mirrors screening approaches (i.e., no use of external calibrators) that are currently employed. Moreover, as discussed herein, these methods often have inherent bias. Finally, PT specimens in 2019 were enriched with acylcarnitines that are nominally isobaric when analyzed by a non-derivatized method. To address this, CDC acylcarnitine QC and PT data were acquired by a derivatized methodology for material certification and reporting purposes. This resulted in data being acquired by two different methods that were used for harmonization, unless otherwise noted, amino acid data were acquired using non-derivatized methodology and acylcarnitine data were acquired using derivatized methodology. It is ideal if data are acquired using a single method for all metabolites for harmonization purposes. Using the CDC non-derivatized methodology for both amino acid and acylcarnitine harmonization yielded similar results as presented in our current study.

### 3.4. Harmonization of Newborn Screening Data in the Future

There are several current studies led by US public health NBS laboratories, with the CDC providing specimens and informatics support. The CDC will distribute blinded specimens and plate maps to participating laboratories with replicate NSQAP QC and PT materials on each plate. This ensures data are acquired under identical conditions and on the same instrument, which should capture a more accurate representation of variation across US public health NBS laboratories and their methods. Current studies include harmonization of methods used for screening adrenoleukodystrophy, lysosomal storage disorders, and amino acid and acylcarnitine disorders. Preliminary results from the adrenoleukodystrophy study—in which long-chain lysophosphatidylcholines were analyzed by mass spectrometry—demonstrate improved comparability of harmonized results across labs when NSQAP QC and PT materials were analyzed on the same plate [39].

The laboratory-centric nature of NBS contributes to the variability in metabolite results, as presented in Table 1 and Figure 2, Figure 3 and Figure 4. The next logical step for harmonizing NBS results would be a paradigm shift toward accuracy, which would facilitate comparability of results across labs and establish disease ranges across populations. Achieving more accurate results could be obtained simply by employing multilevel external calibrators in the NBS workflow. Single-point calibration based on a fixed internal standard concentration is widely used to quantify the metabolites outlined in Table 1 during primary-tier NBS. However, single-point calibration constructs a calibration curve with only two points using the fixed internal standard concentration and forced y-intercept at zero. Therefore, predicted unknown concentrations have more inherent error. One downside of using 6–8 calibrators per plate in an FIA-MS/MS assay is an estimated 8% decrease in throughput, which could be problematic for NBS programs that screen hundreds of thousands of babies per year. Our group and others are currently exploring higher-throughput FIA-MS/MS methods that would offset throughput concerns associated with calibrator adoption. 

**Figure 5:**
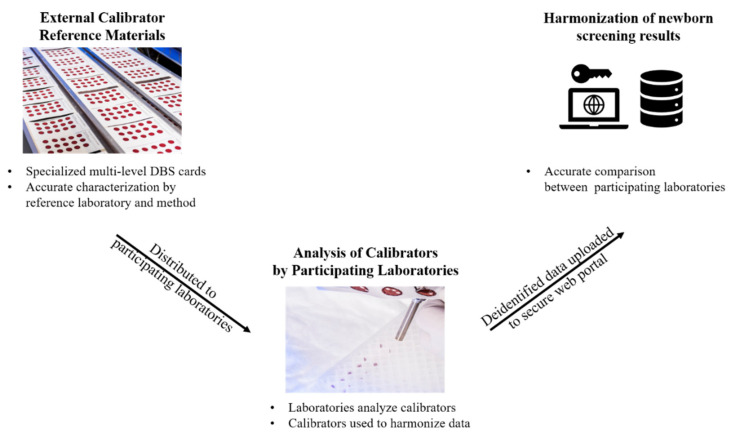
Proof-of-concept future harmonization workflow using external calibration. Harmonizing newborn screening (NBS) data would benefit from the use of external calibrators to increase result accuracy, which would aid comparability of results across laboratories and refining or establishing of disease ranges. The dried blood spot (DBS) calibrators would be distributed to participating laboratories and analyzed alongside clinical samples. Ideally the certified values would reflect the true value of metabolites in the calibrators, thus, potentially correcting for extraction and recovery inefficiencies of the participating laboratories. This de-identified data could be uploaded to a secure web portal to facilitate accurate result comparability across all laboratories.

Currently there are no commercially available DBS-based external calibrators for MS/MS assays. An example NBS workflow for harmonization by external calibration is presented in Figure 5. Specialized DBS materials could be made specifically for external calibration purposes and characterized using a reference method. Ideally, the certified values of metabolites in the DBS calibrators would reflect the true concentration of metabolites, such that extraction and recovery differences across methods could be corrected. Calibrators could be distributed to participating laboratories and analyzed at a specified step in the workflow, such as prior to running a batch of samples or on every plate. The certified calibrator values would be used to create calibration curves and adjust newborn metabolite concentrations from the laboratory. These de-identified newborn data could then be uploaded to a secure centralized web portal that would facilitate the comparison of accurate results and evaluation of cutoffs across participating laboratories. This is one approach that could assist the NBS community to achieve result and cutoff uniformity when appropriate (i.e., similar demographics).

It must be noted that while harmonization is attractive for standardizing disease cutoffs across US public health NBS laboratories, it also poses a difficult challenge since NBS laboratories select their disease cutoffs based on their population demographics and screening method. Some newborn diseases are more prevalent (over 100-fold) in specific geographic locations, especially in consanguineous populations [15,16,17], which contain genetic variants [15,16,17] that may lead to marked differences in the disease range of relevant metabolites. The more logical approach to cutoff harmonization would be to first harmonize NBS methods and results, then use harmonized data to evaluate disease cutoffs in participating laboratories. It is likely that harmonization of NBS results and disease ranges may require several iterations to better understand how different NBS methods and population demographics influence disease cutoffs.

## 4. Conclusions

The purpose of this study was to assess NBS laboratory PT harmonization utilizing retrospectively collected NSQAP participant data from 2019. The harmonization method employed in this study generates regression equations by standardizing an NBS laboratory’s metabolite QC data to a reference laboratory. Overall, these data suggest harmonization of NBS laboratory results using the NSQAP reference materials is a promising approach to help establish cutoffs or achieve cutoff uniformity when appropriate. For instance, a laboratory that begins screening for a new condition, yet has limited access to disease-positive specimens, could establish preliminary cutoffs by using reference materials to harmonize their method to another laboratory. It is important to note that, because it is possible that a reference laboratory may employ a method with low precision, laboratories should use appropriate cautionary measures when replicating this workflow and make informed decisions before using harmonized cutoffs. Our future directions include harmonizing results to expected values (rather than a reference laboratory results), using external calibrators in primary-tier workflows, employing additional statistical models such as a linear random effects model, and using harmonization methods that utilize state-specific data (e.g., multiples of the median, z-score) and do not require a reference laboratory [40,41]. 

## Figures and Tables

**Table 1:** Metabolite descriptive information and results from proficiency test specimens distributed in 2019.

Metabolite (Abbreviation)	Specimen Identifier	Expected Value	CDC PT	Raw PT	Harmonized PT	Number of Labs	Number of Methods
**Arginine (Arg)**	11,954	130.2	125.4	103.9 [65.5–120]	119.32 [96.38–133.95]	21	3
**Free Carnitine (C0)**	41,964	32.52	30.43	26.92 [19.3–36.15]	33.47 [19.69–50.19]	30	5
**Acetylcarnitine (C2)**	41,964	17.15	15.42	14.54 [11.1–19.58]	16.51 [14.3–17.91]	8	4
**Propionylcarnitine (C3)**	31,964	11.04	10.84	9.49 [7.9–15.01]	11.41 [8.38–19.03]	30	5
**Malonylcarnitine (C3DC)**	31,965	25.03	21.82	24.48 [7.02–41.78]	26.8 [23.1–32.55]	6	2
**Butyrylcarnitine (C4)**	31,965	3.04	2.55	2.56 [2.21–4.09]	2.91 [2.21–3.37]	25	5
**Hydroxybutyrylcarnitine (C4OH)**	41,961	3.04	2.68	2.27 [1.65–3.63]	2.96 [2.41–3.9]	6	2
**Isovalerylcarnitine (C5)**	41,965	1.55	1.59	1.4 [1.06–2.48]	1.68 [1.44–1.99]	30	5
**Tiglylcarnitine (C5:1)**	31,965	0.76	0.62	0.48 [0.34–0.8]	0.68 [0.52–1]	24	5
**Hydroxyisovalerylcarnitine (C5OH)**	41,964	1.91	1.71	1.41 [1.1–2.22]	1.89 [1.37–2.29]	29	5
**Glutarylcarnitine (C5DC)**	31,962	1.82	2.10	1.89 [0.51–2.4]	2.26 [1.73–3.13]	29	5
**Hexanoylcarnitine (C6)**	31,963	2.71	2.28	2.35 [1.99–2.73]	2.62 [2.09–4.37]	27	5
**Octanoylcarnitine (C8)**	11,962	0.68	0.69	0.8 [0.64–1.09]	0.78 [0.64–0.95]	27	4
**Decanoylcarnitine (C10)**	31,963	1.95	1.80	1.78 [1.49–2.9]	1.97 [1.63–2.72]	26	5
**Myristoylcarnitine (C14)**	41,962	1.59	1.42	1.55 [1.17–2.08]	1.65 [1.42–1.96]	26	5
**Tetradecenoylcarnitine (C14:1)**	41,962	1.75	1.29	1.27 [0.93–1.95]	1.45 [1.16–1.72]	26	5
**Palmitoylcarnitine (C16)**	31,965	15.52	11.44	12.12 [10.43–14.34]	12.62 [10.58–15.68]	27	5
**Hydroxypalmitoylcarnitine (C16OH)**	41,963	1.01	0.81	0.69 [0.52–1.08]	1.01 [0.77–1.24]	29	5
**Stearoylcarnitine (C18)**	11,965	3.77	4.22	3.36 [2.8–3.74]	3.42 [3.12–3.77]	23	4
**Hydroxystearoylcarnitine (C18OH)**	41,963	0.80	0.44	0.55 [0.47–0.74]	0.57 [0.43–0.72]	21	5
**Citrulline (Cit)**	11,951	181.3	190.3	180.7 [117.4–214]	187.1 [130.9–254.3]	27	4
**Leucine (Leu)**	41,951	450.0	521.3	498.8 [338.9–621.1]	496.4 [436–571]	30	5
**Methionine (Met)**	41,955	185.0	140.6	149.2 [116–188]	176.4 [129.43–215.83]	30	5
**Phenylalanine (Phe)**	11,952	311.4	296	295.9 [32–1685.4]	338.42 [23.8–1743.5]	268	16
**Succinylacetone (SUAC)**	41,953	50.0	28.2	17.6 [10.1–53.4]	26.6 [12.5–49.9]	26	4
**Tyrosine (Tyr)**	41,953	900.0	930.8	859.6 [558.3–1228]	894.3 [749.7–1046.4]	27	5
**Valine (Val)**	41,951	450.0	473.8	492.7 [266.9–639.3]	463.6 [369.8–540.4]	20	5

Amino acid and acylcarnitine metabolites and the corresponding specimen identifier used in this study are presented from the 2019 proficiency test (PT). Each metabolite’s expected value and CDC PT value are presented in µM units. The CDC PT values reported in 2019 were from our derivatized method. Raw and harmonized PT values are presented in µM units in the format of mean (minimum–maximum). All data are from US public health newborn screening (NBS) laboratories, except phenylalanine data which also includes US non-public health and international NBS laboratories.

## Supplementary Materials

The following are available online at https://www.mdpi.com/2409-515X/6/3/75/s1. Supplementary Figure S1. Distribution of regression equation slopes from each metabolite.

## Abbreviations

C3	Propionylcarnitine
C4OH	Hydroxybutyrylcarnitine
C5DC	Glutarylcarnitine
C8	Octanoylcarnitine
C16	Palmitoylcarnitine
C18	Stearoylcarnitine
CDC	The Centers for Disease Control and Prevention
DBS	Dried blood spot
FIA	Flow injection analysis
IEM	Inborn errors of metabolism
LDT	Laboratory developed test
MS/MS	Tandem mass spectrometry
NBS	Newborn screening
NSQAP	Newborn Screening Quality Assurance Program
Phe	Phenylalanine
PT	Proficiency testing
QA	Quality assurance
QC	Quality control
SUAC	Succinylacetone
Tyr	Tyrosine
