# Supplementary material for: Harmonizing Newborn Screening Laboratory Proficiency Test Results Using the CDC NSQAP Reference Materials
Source: Int J Neonatal Screen. 2020 Sep 17;6(3):75. doi: 10.3390/ijns6030075 (PMC7570198; doi:10.3390/ijns6030075)
Supplement: Supplementary file 1 [file IJNS-06-00075-s001.pdf]

Supplementary Figure 1. Distribution of regression equation slopes from each metabolite

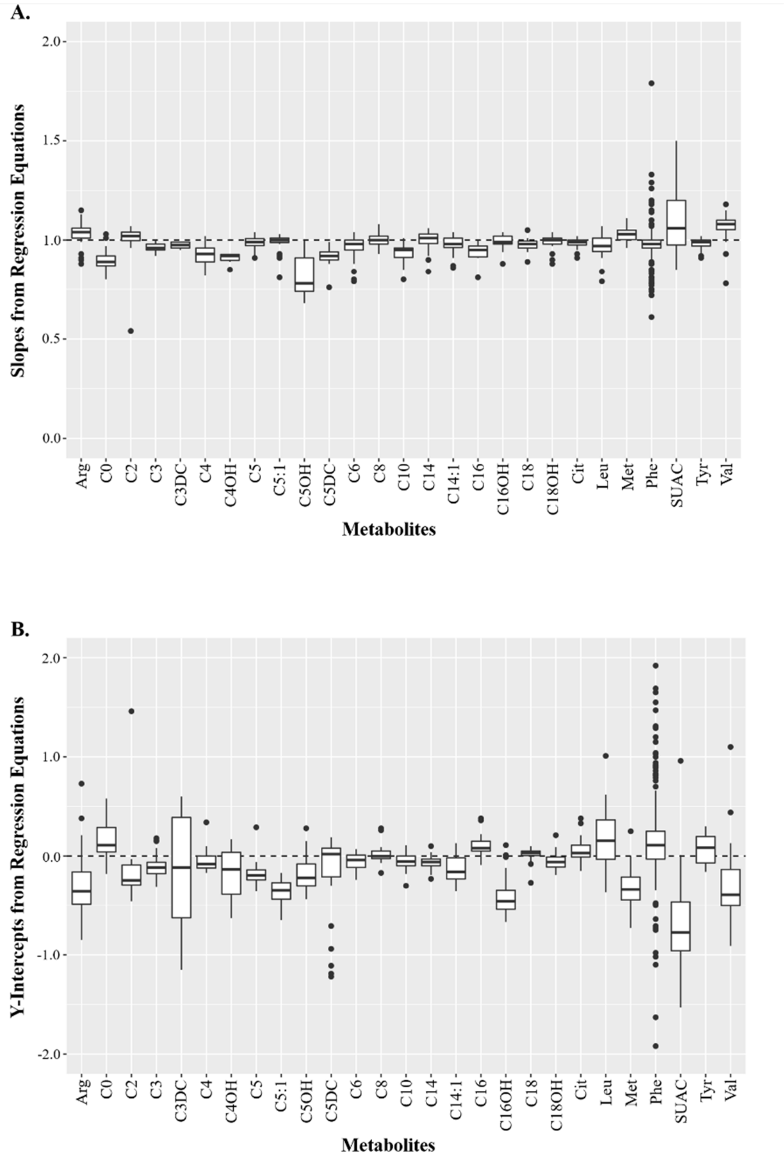

Quality control data reported by participating laboratories were used to generate regression equations, for each laboratory and metabolite, using quality control data reported by the Center for Disease Control and Prevention's Biochemical Mass Spectrometry Laboratory. Slope (A) and y-intercept (B) regression parameters generated throughout our study are displayed. Clearly, if a laboratory's slope was exactly one and the intercept was exactly zero, the laboratory's QC measurements would have been very similar to the CDC QC measurements. As a result, their harmonized PT would be identical to their raw PT measurement. The majority of the analytes have laboratory slopes less than one (A) and intercepts around zero (B). Since the QC data are log transformed prior to regression analysis, any type of proportional bias from the CDC QC measurements is captured in the y-intercept. Box plots were constructed from regression equation slopes across participating laboratories. All data are from domestic State newborn screening laboratories, except phenylalanine data which also include domestic non-State and international newborn screening laboratories.
